# Lonafarnib Inhibits Farnesyltransferase via Suppressing ERK Signaling Pathway to Prevent Osteoclastogenesis in Titanium Particle-Induced Osteolysis

**DOI:** 10.3389/fphar.2022.848152

**Published:** 2022-03-01

**Authors:** Linke Huang, Weiwei Chen, Linhua Wei, Yuangang Su, Jiamin Liang, Haoyu Lian, Hui Wang, Feng Long, Fan Yang, Shiyao Gao, Zhen Tan, Jiake Xu, Jinmin Zhao, Qian Liu

**Affiliations:** ^1^ Research Centre for Regenerative Medicine, Orthopaedic Department, The First Affiliated Hospital of Guangxi Medical University, Nanning, China; ^2^ Department of Orthopaedics, The Second Affiliated Hospital of Guangxi Medical University, Nanning, China; ^3^ Guangxi Key Laboratory of Regenerative Medicine, Guangxi Medical University, Nanning, China; ^4^ The Affiliated Nanning Infectious Disease Hospital of Guangxi Medical University, The Fourth People’s Hospital of Nanning, Nanning, China; ^5^ School of Biomedical Sciences, University of Western Australia, Perth, WA, Australia

**Keywords:** lonafarnib, farnesyltransferase, ERK, osteoclast, osteolysis

## Abstract

Wear debris after total joint arthroplasty can attract the recruitment of macrophages, which release pro-inflammatory substances, triggering the activation of osteoclasts, thereby leading to periprosthetic osteolysis (PPOL) and aseptic loosening. However, the development of pharmacological strategies targeting osteoclasts to prevent periprosthetic osteolysis has not been fruitful. In this study, we worked toward researching the effects and mechanisms of a farnesyltransferase (FTase) inhibitor Lonafarnib (Lon) on receptor activator of nuclear factor κB (NF-κB) ligand (RANKL)-induced osteoclastogenesis and bone resorption, as well as the impacts of Lon on titanium particle-induced osteolysis. To investigate the impacts of Lon on bone resorption and osteoclastogenesis *in vitro*, bone marrow macrophages were incubated and stimulated with RANKL and macrophage colony-stimulating factor (M-CSF). The influence of Lon on osteolysis prevention *in vivo* was examined utilizing a titanium particle-induced mouse calvarial osteolysis model. The osteoclast-relevant genes expression was explored by real-time quantitative PCR. Immunofluorescence was used to detect intracellular localization of nuclear factor of activated T cells 1 (NFATc1). SiRNA silence assay was applied to examine the influence of FTase on osteoclasts activation. Related signaling pathways, including NFATc1 signaling, NF-κB, mitogen-activated protein kinases pathways were identified by western blot assay. Lon was illustrated to suppress bone resorptive function and osteoclastogenesis *in vitro*, and it also reduced the production of pro-inflammatory substances and prevented titanium particle-induced osteolysis *in vivo*. Lon decreased the expression of osteoclast-relevant genes and suppressed NFATc1 nuclear translocation and auto-amplification. Mechanistically, Lon dampened FTase, and inhibition of FTase reduced osteoclast formation by suppressing ERK signaling. Lon is a promising treatment option for osteoclast-related osteolysis diseases including periprosthetic osteolysis by targeted inhibition of FTase through suppressing ERK signaling.

## Introduction

Bone homeostasis relies on the exquisite balancing act between osteoblastic bone formation and osteoclastic bone resorption. Overactivation of osteoclasts can result in a variety of bone illnesses, including postmenopausal osteoporosis, periprosthetic osteolysis (PPOL), and rheumatoid arthritis. When osteoclasts form from the monocyte/macrophage hematopoietic lineage, they exhibit tartrate-resistant acid phosphatase (TRAP)-positive multinucleated cells that have an impact on bone resorption (Boyle et al., 2003). The growth and differentiation of osteoclast are modulated by two critical cytokines, named receptor activator of nuclear factor-kappa B (NF-κB) ligand (RANKL) and macrophage colony-stimulating factor (M-CSF) (Tobeiha et al., 2020). RANKL mediates the stimulation of many early signal transduction pathways, including the NF-κB pathway (Yang et al., 2021) and mitogen-activated protein kinases (MAPKs) pathway (Hou et al., 2005), ultimately resulting in the activation of the nuclear factor of activated T cells 1 (NFATc1) in addition to c-Fos (Boyle et al., 2003; Tobeiha et al., 2020). Such signaling cascades activate the expression of typical osteoclast genes such as *Dcstamp* (encoding dendritic cell-specific transmembrane protein), *Ctsk* (encoding Cathepsin K), *Acp5* (encoding tartrate-resistant acid phosphatase [TRAP]), and *Mmp9* (encoding matrix metalloproteinase 9), thus ultimately triggering osteoclast differentiation, activation, and survival (Boyle et al., 2003).

Primary total joint arthroplasty (TJA) is among the most prevalent orthopedic procedures for end-stage joint illnesses, with more than three million TJA registered in the world each year (Ben-Shlomo et al., 2020). The majority of patients have wonderful outcomes, with relieving pain and improving function. Despite advances in mechanical research, prosthesis coating materials, and surgical techniques, and several studies reported a greater than 90% survivorship at 15–20 years after TJA (Badarudeen et al., 2017; Carter et al., 2019), aseptic loosening (AL) is among the major causes of failure (Sadoghi et al., 2013; Sharkey et al., 2014). It is estimated that 55% of hip (Sadoghi et al., 2013) and 31% of knee (Sharkey et al., 2014) revisions are caused by AL accompanied by PPOL. Wear particles after TJA, such as polyethylene, titanium, and polymethyl methacrylate, can attract the recruitment of macrophages along with other cells, which produce chemokines, cytokines, and other pro-inflammatory components that sustain chronic inflammation, inducing osteoclastic differentiation and bone resorption (Goodman and Gallo, 2019; Zhang et al., 2020). Furthermore, it’s reported that these factors have been investigated under the periprosthetic milieu (Masui et al., 2005). Hence, repression of osteoclastogenesis and pro-inflammatory production is a potential therapy to protect against wear particle-induced PPOL.

Farnesyltransferase (FTase) is responsible for catalyzing the farnesylation of proteins, which performs an instrumental function during the RhoB and Ras families activation (Fritz and Kaina, 2006; Delarue et al., 2007). Prenylation of the Ras protein (geranylgeranylation and farnesylation) is shown to have a function in a variety of illnesses, such as bone mineralization disorders (Itzstein et al., 2011), progeria (Young et al., 2013), inflammatory diseases (Wang and Casey, 2016), glaucoma (Rao et al., 2008), neurological diseases (Li et al., 2016), and cancer (Berndt et al., 2011). FTase is a heterodimer consisting of an α-subunit (alpha, CAAX box, and farnesyltransferase [FNTA]) and a β-subunit (beta, CAAX box, and farnesyltransferase [FNTB]) (Schaeffer et al., 2016). During the catalytic cycle of an enzyme, active centers are created by an overlap between the central core of the α-subunit and the portion of the β-subunit. The discovery that the farnesylation of Ras protein, which is shown to be instrumental for the normal functioning of Ras, attracted the attention for a search of specific FTase inhibitors. Lonafarnib (Lon), an active FTase inhibitor, is the only approved drug for Hutchison-Gilford progeria syndrome (Dhillon, 2021). A low dose of Lon is a promising therapy for Hepatitis delta virus (Guo and King, 2015). Sun et al. (2015) reported that Lon has a protective effect on atherosclerosis by inhibiting plaque neovascularization. Furthermore, many studies demonstrated the pleiotropic effect of Lon, such as the anti-inflammatory activity (Takada et al., 2004), and anti-tumor effects (Wang et al., 2017). Recently, research reports have indicated that Lon had potential combat to COVID-19 (Santos-Beneit et al., 2021). However, the role of FTase in osteoclasts, and the effects and mechanisms of Lon suppressing FTase on bone metabolism, including the differentiation and function of osteoclasts, remain unclear.

Herein, we evaluated the effects of Lon suppressing FTase on RANKL-induced osteoclastic differentiation and osteoclastic bone resorption, and further elucidated its mechanisms. Moreover, we investigated the protective effect of Lon in a titanium particle-induced mouse calvarial osteolysis model.

## Materials and Methods

### Antibodies and Reagents

Lon was supplied by APExBIO (Houston, Texas, United States, Catalog No. A4379), and its purity is over 98%. Alpha-modified minimum essential medium (α-MEM), fetal bovine serum (FBS), and penicillin-streptomycin solution were provided by Gibco (Thermo Fisher Scientific, Waltham, MA, the United States). Cell Counting Kit-8 (CCK-8) was purchased from MedChemExpress (MCE, Shanghai, China). Lon was dissolved into a storage concentration of 20 mM with dimethyl sulfoxide (DMSO), followed by the addition of α-MEM to dilute Lon to various working concentrations. Recombinant mouse RANKL and recombinant mouse M-CSF were obtained from R&D Systems (Minneapolis, MN). The Cell Signaling Technology (Danvers, MA, the United States) supplied the primary antibodies (anti-P65, anti-p-P65, anti-IκB-α, anti-β-actin, anti-P38, anti-p-P38, anti-ERK, anti-p-ERK, anti-JNK, and anti-p-JNK) and secondary antibodies (mouse and rabbit). The antibodies (NFATc1, c-Fos, and Cathepsin K) were purchased from Abcam (Cambridge, the United Kingdom). FNTA polyclonal antibody was supported by WUHAN SANYING (Wuhan, Hubei, China). NFATc1 Antibody Alexa Fluor® 488 was supplied by Santa Cruz Biotechnology (Dallas, CA, United States).

### Titanium Particle Preparation

Johnson Matthey Company (Ward Hill) provided the titanium particles with diameters ranging from 0.27 to 2.04 μm. To eliminate endotoxin from the particles, they were immersed for 96 h in a solution of 75% (v/v) ethanol. The endotoxin level of titanium particles was assessed by Limulus amoebocyte lysate assay, and the endotoxin unit (EU) concentration of less than 0.1 EU/ml was considered acceptable. Titanium particles were mixed with sterile phosphate buffer (PBS) for a 200 mg/ml concentration.

### Experimental Animals

C57BL/6J mice were procured from the Animal Laboratory Centre of Guangxi Medical University. The animals were maintained under standard conditions (in a chamber maintained at a temperature ranging between 22 and 24°C and humidity ranging between 55 and 60% and a 12/12 h darkness/light cycle) with an adequate supply of food and water and acclimatized for 1 week. The Animal Ethics Committee of Guangxi Medical University (Nanning, Guangxi, China) granted its approval for all animal experiments in the present research.

### Titanium Particle-Induced Osteolysis Model and Experimental Protocol

The Animal Experiment Centre of Guangxi Medical University granted its approval for the titanium particle-induced mouse calvarial osteolysis experiment. The experiment randomized 24 healthy male C57BL/6J mice (8-week-old) into four groups as follows (*n* = 6 for each group): Sham group (PBS local injection only), Vehicle group (titanium particles with local injection), Lon (0.25 mg/kg) group (titanium particles with 0.25 mg/kg Lon topical injection), Lon (0.5 mg/kg) group (titanium particles with 0.5 mg/kg Lon topical injection). The surgical procedure was conducted as described (Qin et al., 2012). Briefly, the cranial skin and periosteum of mice were incised after anesthetization. Before suturing the cranial skin and periosteum together, a sum of 20 mg titanium particles in PBS was inserted beneath the periosteum. Subperiosteal injection of Lon in PBS or PBS alone proceeded each day for 2 weeks. Subsequently, the mice were euthanized, and all of their calvaria were taken and subjected to micro-computed tomography (micro-CT) scanning, as well as histologic evaluation.

### Micro-CT Scanning

The calvaria were fixed with 4% PFA. Titanium particles were removed from calvaria before micro-CT scanning to avoid metal artifacts. The fibrous membranes along with the titanium particles were gently peeled from the surface of the calvaria using a periosteum detacher. A Skyscan 1176 micro-CT system (Bruker, Billerica, MA, United States) was used to scan the calvaria at a voltage of 80 kV and a current of 80 μA with an isometric pixel size of 9 mm. The SkyScan NRecon platform was used to rebuild three-dimensional (3-D) pictures, then subjected to examination utilizing SkyScan CTAn software (Bruker). A region of interest (ROI) of square shape surrounding the midline sutures of the calvaria was chosen for additional quantitative and qualitative investigations. As previously indicated, the criteria under consideration comprise the percentage of bone volume/tissue volume (BV/TV), the percentage of total porosity, and the number of porosities (Bouxsein et al., 2010).

### Histological Analysis

Histomorphometry analyses were processed following the methods of Dempster et al. (Dempster et al., 2013). After all, samples were scanned, the calvaria were decalcified with 10% EDTA decalcified solution at a temperature of 4°C for 3 weeks and subsequently embedded in paraffin. 5-mm thickness bone segments were subjected to hematoxylin and eosin (H&E), TRAP, and immunohistochemical staining. The images were obtained using a KFPRO scanner (KONFOONG BIOTECH INTERNATIONAL CO., LTD. Ningbo, China), and visualized by K-Viewer software (KONFOONG BIOTECH INTERNATIONAL CO.). Osteoclasts were defined as multinucleated TRAP-positive cells that were found in close proximity to bone. With the aid of ImageJ Software in a blinded and nonbiased way, two researchers were able to examine bone histomorphometry independently. To determine the amount of erosion surface present per sample, we normalized the number of TRAP-positive multinucleated osteoclasts by the area of the bone, followed by the assessment of the proportion of erosion surface per sample.

### Isolation of Macrophages From Mouse Bone Marrow

Six-week-old mice bone marrow macrophages (BMMs) were separated by flushing out the marrow cavities of their tibias and femurs. BMMs are fusiform and grow by adhering to the wall, and M-CSF can promote their survival and proliferation. Subsequently, culturing of BMMs was performed in complete α-MEM (comprising 1% Penicillin-streptomycin solution, 25 ng/mL M-CSF, 10% FBS) for 3 days. The adhesion cells were used in the following various experiments.

### Osteoclasts Culture *In Vitro*

In 96-well plates, BMMs (6 × 10^3^ cells/well) were subjected to incubation overnight at a temperature of 37°C in a humidity chamber comprising 5% CO_2_ and 95% air. To induce BMM fusion, the medium was renewed every 2 days while the cells were cultured in complete α-MEM incorporating RANKL (50 ng/ml) in the presence of a range of Lon concentrations (0, 0.125, 0.25, 0.5 and 1.0 μM). To further explore the time effects of Lon on osteoclastogenesis, BMMs were incubated with 0.5 μM Lon at 3 phases (initial phase: the first to third day; middle phase: the third to fifth day; later phase: the fifth to seventh day) under RANKL activation. For the purpose of visualizing TRAP-positive multinucleated cells, the cells were first fixed for 10 min in a 4% paraformaldehyde (PFA) solution, followed by the staining of the TRAP reagent. If the numbers of cell nuclei were equal or more than three, the cells were categorized as osteoclasts and then subjected to counting. The stained images were photographed by Cytation 5 (BioTek Instrument Incorporation, Winooski, VT, the United States).

### Cell Viability Assay

To test the cytotoxicity of Lon on BMMs, a CCK-8 assay was conducted. Seeding of BMMs was carried out in 96-well plates at a density of 6 × 10^3^ cells per well and incubated overnight. Then, the complete culture medium was replaced, and incubation of the cells was conducted at diverse concentrations of Lon in the culture medium for 48 h. Then, 100 μl of new medium containing 10 μl of CCK8 was added and subjected to incubation for 2 h at a temperature of 37°C Absorbance was read with a TriStar2 LB 942 Multimode Microplate Reader (Berthold Technologies Gmbh & Co. KG, Baden-Württemberg, Germany) at 450 nm.

### Podosome Actin Belt Formation Assay

The actin cytoskeleton of osteoclasts was examined utilizing Podosome actin belt formation assay to probe into if Lon may have an effect on it. Following the aforementioned procedure, BMMs were induced into osteoclasts in the presence or absence of Lon (0, 0.25, 0.5 μM). A solution of 0.1% Triton X-100 in PBS was used to incubate the cells for 3 min after being fixed for 10 min with 4% PFA. After that, the samples were dyed at room temperature in a dark condition with rhodamine-phalloidin and DAPI for 1 h and 5 min, respectively. Podosome actin belt and nuclei were visualized by Cytation 5.

### Osteoclast Acidification Assay

Acridine orange dye (3,6-bis [Dimethylamine] acridine) was used to evaluate the influence of Lon on the acidification in osteoclasts as in the previous study (Sørensen et al., 2007; Qin et al., 2011). Briefly, the induction of BMMs was achieved using RANKL stimulation in the presence or absence of Lon (0, 0.25, 0.5 μM) until osteoclast-like cells appeared. The cells were incubated in α-MEM with acridine orange at 5 μg/ml for 15 min. Then, the dye was washed away by PBS. Images were taken using laser confocal microscopy (Carl Zeiss Meditec, Jena, Germany) at an emission of 535 nm and excitation of 492 nm.

### Bone Pits Resorption Assay

To investigate whether Lon affects osteoclast bone resorption function, the bone pits resorption assay was conducted as reported (Wang et al., 2020). BMMs were plated in 6-well plates in complete α-MEM at a 1 × 10^5^ cells/well density. After incubating overnight, a concentration of 50 ng/ml RANKL was used to activate cells until small osteoclasts appeared. Then small osteoclasts were then gently detached and collected. The same number of small osteoclasts was seeded into each well of the bone piece for bone pits resorption assay. Treatment of the cells was performed with increasing dosages of Lon (0, 0.25, 0.5 μM) in complete α-MEM that contained 50 ng/ml RANKL. After replacing the culture medium twice, bone pieces were washed with PBS to remove superficial cells as much as possible. The images of bone pits resorption areas were acquired at a magnification of 3.0 kV utilizing a Quanta 250 scanning electron microscope (SEM; FEI; Thermo Fisher Scientific, Inc), followed by quantification utilizing Image J software (NIH, Bethesda, Maryland, United States). To count the osteoclasts, the residual samples were fixed with 4% PFA and subjected to staining for TRAP activity as described in the previous section.

### FTase Silence by SiRNA

Sangon Biotech (Shanghai, China) developed a siRNA that targets the α-subunit of FTase for the silencing of the FTase gene expression. Transfection of negative control siRNA (NC siRNA) or special FTase siRNA into BMMs was performed utilizing RNATransMate (Sangon Biotech, Shanghai, China) in accordance with the guidelines stipulated by the manufacturer. Briefly, BMMs were cultured in plates for one night. Then, the α-MEM medium containing RNATransMate-siRNA compound was added for transfection. A new medium that contained 10% FBS was added following 6 h. To determine the efficiency of transfection, fluorescence-labeled siRNA was transfected into BMMs as a control. The efficacy of transfection was evaluated using a fluorescent microscope 24 h later. The siRNA oligonucleotide for FTase has the following sequence: 5-CAG​AGC​AGA​AUG​GGC​UGA​UAU​TT-3, the antisense is 5-AUA​UCA​GCC​CAU​UCU​GCU​CUG​TT-3. The sense of NC siRNA is 5-UUC​UCC​GAA​CGU​GUC​ACG​UTT-3, the antisense is 5-ACG​UGA​CAC​GUU​CGG​AGA​ATT-3.

### Real-Time Quantitative PCR (QT-PCR)

Incubation of BMMs was performed in a 6-well plate, followed by the induction of the cells by RANKL with various Lon (0, 0.125, 0.25, 0.5 μM) until osteoclasts appeared. TRIzol (Thermo Fisher Scientific) was utilized to extract the total RNA of each sample. Thereafter, total RNA was reversed transcription into complementary DNA (cDNA). Following that, the acquired cDNA was employed as a QT-PCR template on the LightCycler® 96 system (Roche, Basel, Switzerland). The special primers used in this research were presented in Table 1. The following were the cycle conditions for the PCR reaction: denaturation for 10 min at a temperature of 95°C, proceeded by 55 cycles with a similar temperature for 15 s, proceeded by 15 s at 60°C, and lastly 40 s at 72°C. The 2^−ΔΔCT^ method was applied to analyze the data, and the normalization of the expression of the target gene to the average expression of *β-actin* was performed.

**TABLE 1:** The primer sequences of the osteoclast-related genes used in QT-PCR.

Gene	Forward primer (5ʹ–3ʹ)	Reverse primer (5ʹ–3ʹ)
*Acp5*	TGTGGCCATCTTTATGCT	GTCATTTCTTTGGGGCTT
*Nfatc1*	GGT​GCT​GTC​TGG​CCA​TAA​CT	GAA​ACG​CTG​GTA​CTG​GCT​TC
*Ctsk*	AGG​CGG​CTC​TAT​ATG​ACC​ACT​G	TCT​TCA​GGG​CTT​TCT​CGT​TC
*Mmp9*	GAA​GGC​AAA​CCC​TGT​GTG​TGT​T	AGA​GTA​CTG​CTT​GCC​CAG​GA
*Dcstamp*	TCT​GCT​GTA​TCG​GCT​CAT​CTC	ACTCCTTGGGTTCCTTGCTT
*c-fos*	CCA​GTC​AAG​AGC​ATC​AGC​AA	AAG​TAG​TGC​AGC​CCG​GAG​TA
*β-actin*	TCT​GCT​GGA​AGG​TGG​ACA​GT	CCT​CTA​TGC​CAA​CAC​AGT​GC

### Immunofluorescence

In the present research, we conducted the Immunofluorescent identification of intracellular NFATc1 in accordance with the procedure as explained previously (Hasegawa et al., 2010; Park et al., 2018) with some modifications. Briefly, BMMs were plated at a density of 5 × 10^4^ cells/dish in confocal dishes, cultured overnight, and treated with or without 0.5 μM Lon for 48 h in complete α-MEM that contained 50 ng/ml RANKL. Once the fixation with 4% PFA had been completed for 10 min at ambient temperature in the darkness, 0.1% Triton X-100 was employed to permeabilize the cells for 3 min, followed by an hour of blocking with 3% bovine serum albumin (BSA). After rinsing using PBS, the fixed cells were subjected to incubation by adding NFATc1 Antibody Alexa Fluor® 488 at a dilution of 1:200 over the night at a temperature of 4°C and then subjected to counterstaining with DAPI for 5 min. Images were photographed using laser confocal microscopy (Leica STELLARIS, Heidelberg, Germany). NFATc1 level in nuclei fraction was counted using ImageJ software as reported (Liu et al., 2019).

### Western Blot

BMMs were seeded at a density of 1 × 10^6^ cells/well in 6-well plates, followed by stimulation using M-CSF and RANKL with or without Lon of designated dosages for the set times or the specified time for various concentrations. To extract proteins, we utilized the radioimmunoprecipitation (RIPA) lysis buffer to split the treated cells. SDS-polyacrylamide gel electrophoresis (SDS-PAGE) was utilized to isolate the proteins, which were then deposited to a nitrocellulose membrane (Thermo Fisher Scientific, Shanghai, China). After that, the nitrocellulose membranes were infiltrated in 5% skim milk to block non-specific binding immunoreactivities. Subsequently, the membranes were subjected to incubation overnight at a temperature of 4°C with primary antibodies (1:1,000) and were gently shaken. On the following day, membranes were rinsed 3 times utilizing Tris-buffered saline plus 0.1% Tween 20 (TBST), followed by incubation using corresponding anti-mouse or anti-rabbit antibodies for 1 hour at ambient temperature. The images were captured with the aid of an ImageQuant LAS-4000 device (GE Healthcare, Chicago, Illinois, United States) and processed utilizing the ImageJ.

### Statistical Analysis

Each experimental results presented were representative of equal or more than three independent experiments, and data are articulated as mean ± standard deviation. The data were evaluated statistically by One-way ANOVA or Student’s *t*-test, followed by Tukey’s *post hoc* analysis, if appropriate. The differences were deemed statistically significant once the *p*-value recorded was below 0.05.

## Results

### Lon Inhibits RANKL-Induced Osteoclastogenesis *In Vitro*

**FIGURE 1:**
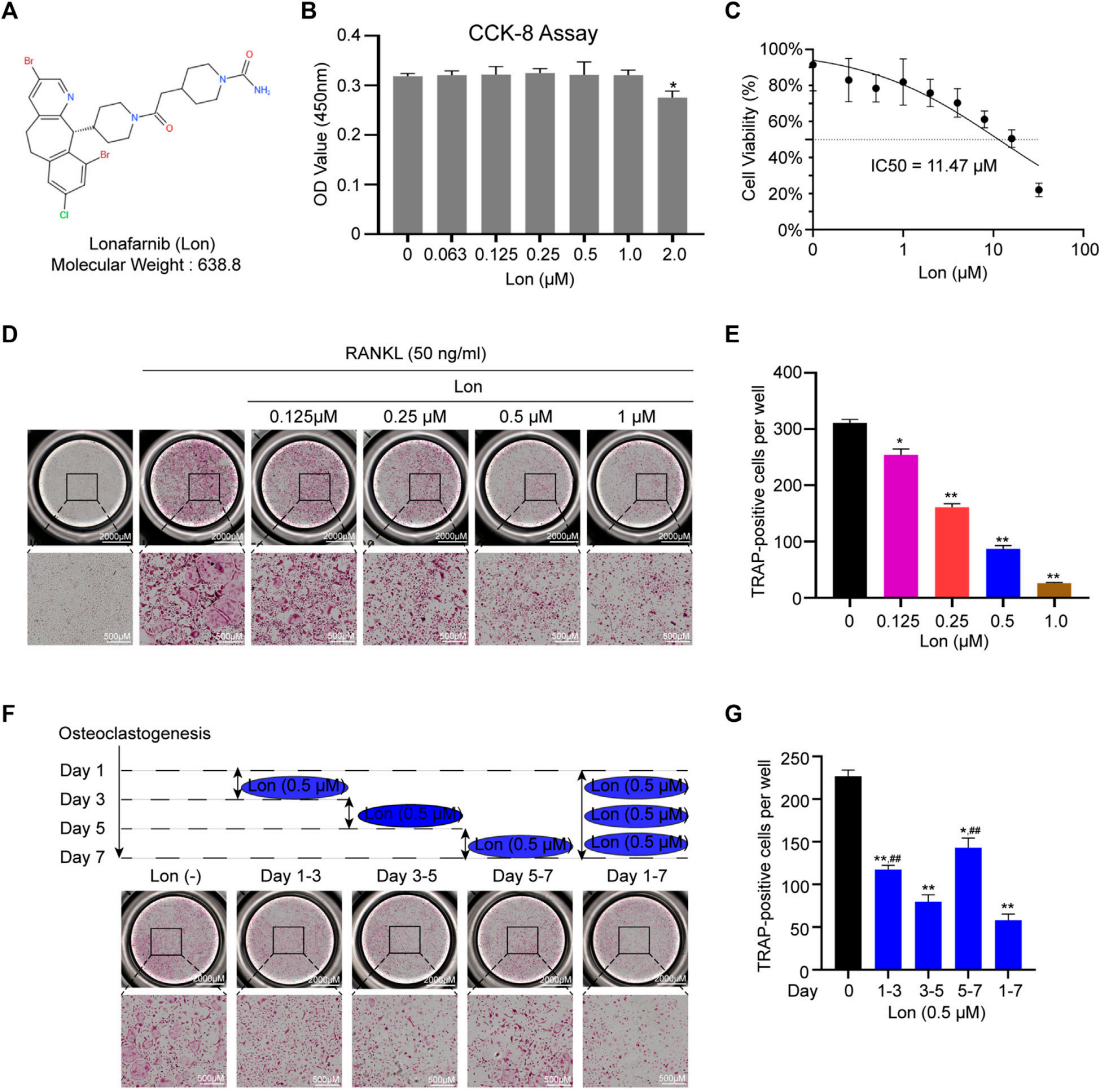
Lon suppresses RANKL-induced osteoclastogenesis *in vitro*. **(A)** The molecular structure and weight of Lon. **(B)** The cytotoxicity of Lon against BMMs at 48 h after treatment was investigated by CCK-8 assay. **(C)** IC50 for Lon against BMMs at 48 h was calculated. **(D)** Representative images of TRAP staining showing that Lon inhibited osteoclastogenesis in a dose-dependent manner. **(E)** Quantification of multinucleated TRAP-positive osteoclast (nuclei ≥ 3). **(F)** Representative images of TRAP staining showing that BMMs treated with Lon (0.5 μM) in the certain time stages during osteoclastogenesis. **(G)** Quantification of multinucleated TRAP-positive osteoclast (nuclei ≥ 3) treated with Lon in the certain time stages. All of the above data are expressed as the mean ± SD, *n* = 3 per group, *p < 0.05, **p < 0.01 compared with control group (without Lon treatment). #*p* < 0.05, ##*p* < 0.01 compared with the middle phase. BMMs, bone marrow macrophages; IC50, the half maximal inhibitory concentration; Lon, Lonafarnib; OD, optical density; RANKL, receptor activator of nuclear factor-κB ligand; TRAP, tartrate-resistant acid phosphatase.

Figure 1A shows the molecular structure and weight of Lon. The cytotoxicity of Lon against BMMs was investigated using a CCK-8 test 48 h following the initiation of the treatment. We found that Lon didn’t inhibit the proliferation of BMMs when the concentration was less than 1 μM (Figure 1B). The half-maximal inhibitory concentration (IC50) for Lon against BMMs at 48 h was calculated to be 11.47 μM (Figure 1C). BMMs were induced using RANKL with or without Lon (0.125, 0.25, 0.5, and 1 μM), and the results were used to determine if Lon had a suppressive impact on osteoclastogenesis. The TRAP results indicated that Lon treatment showed a dose-dependent suppression of osteoclast formation. The numbers and size of multinucleated TRAP-positive osteoclast were remarkably reduced as the Lon dose was added (Figures 1D,E). To determine which stage of osteoclast differentiation was influenced, cells were performed with 0.5 μM Lon at different certain time stages (Figure 1F). Lon affected all stages of osteoclast differentiation. However, it mainly played its repressive role during the middle phase (Day 3–5) of osteoclastogenesis, instead of the initial phase (Day 1–3) (Figure 1G).

### Lon Influences the Bone Resorptive Function of Osteoclasts *In Vitro*

**FIGURE 2:**
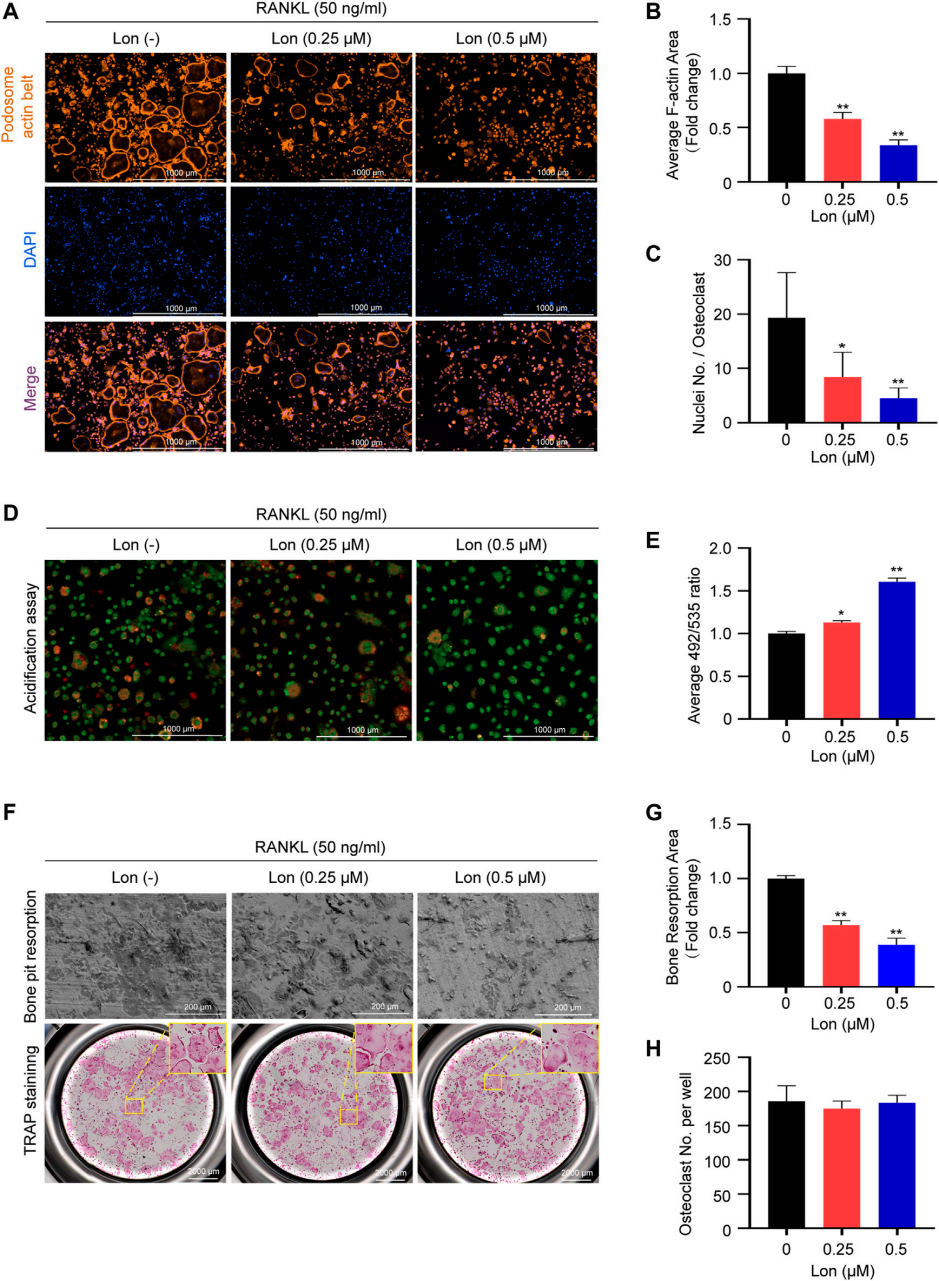
Lon affects the bone resorptive function of osteoclasts *in vitro*. **(A)** Representative images showing the impaired podosome belts formation in osteoclasts treated with the indicated concentrations of Lon. **(B)** Quantification of the relative area of osteoclast (*n* = 3 per group). **(C)** Quantification of the nuclei number per osteoclast (*n* = 10 per group). **(D)** Representative confocal microscopy images showing the impaired the acidification in osteoclasts treated with the indicated concentrations of Lon. **(E)** Quantification of acidification mediated quenching of acridine orange (*n* = 3 per group). **(F)** Representative images showing the osteoclastogenesis and bone pits resorption in each group. Osteoclasts were seeded in bone pieces and treated by RANKL the indicated concentrations of Lon. Half of the wells for each group were stained by TRAP. **(G)** Quantification of relative resorbed bone piece area per well in each group (*n* = 3 per group). **(H)** Quantification of the numbers of osteoclasts per well in each group (*n* = 3 per group). All of the above data are expressed as the mean ± SD. *p < 0.05, **p < 0.01 compared with control group (without Lon treatment). Lon, Lonafarnib; RANKL, receptor activator of nuclear factor-κB ligand; TRAP, tartrate-resistant acid phosphatase.

Podosome actin belt formation reflects the functional state and the cytoskeletal integrity of osteoclasts (Wilson et al., 2009). We performed rhodamine-phalloidin staining to investigate the potential effects of Lon on osteoclast morphological changes and podosome actin belt formation. The results manifested that well-defined podosome actin belts containing complete nuclei in mature osteoclasts after RANKL induction. However, the size of osteoclasts and the numbers of osteoclastic nuclei were obviously decreased after treatment with Lon (0.25, 0.5 μM) (Figures 2A–C).

As shown in Figure 2D, osteoclasts showed orange-red fluorescence after RANKL stimulation, indicating active production of H^+^. Treatment with Lon dose-dependently (0.25, 0.5 μM) inhibited acidification. In order to quantify acidification, we measured quenching of acridine orange regulated by acidification. Figure 2E shows that Lon inhibited acidification in a dosage-dependent way, culminating in lesser quenching and subsequent greater levels of green fluorescence.

Next, we investigated whether Lon had any effect on osteoclast resorption function using bone pit resorption assay. The addition of Lon reduced bone resorptive area (Figure 2F). Compared with the control group, a dosage-dependent repressive impact of Lon on osteoclastic resorptive activity was detected (Figure 2G). Osteoclasts did not alter in number (Figures 2F,H), indicating that the effect of Lon on osteoclastic resorptive function is not due to the increase in the count of osteoclasts.

### Lon Reduces Titanium Particle-Induced Bone Osteolysis in Mice

**FIGURE 3:**
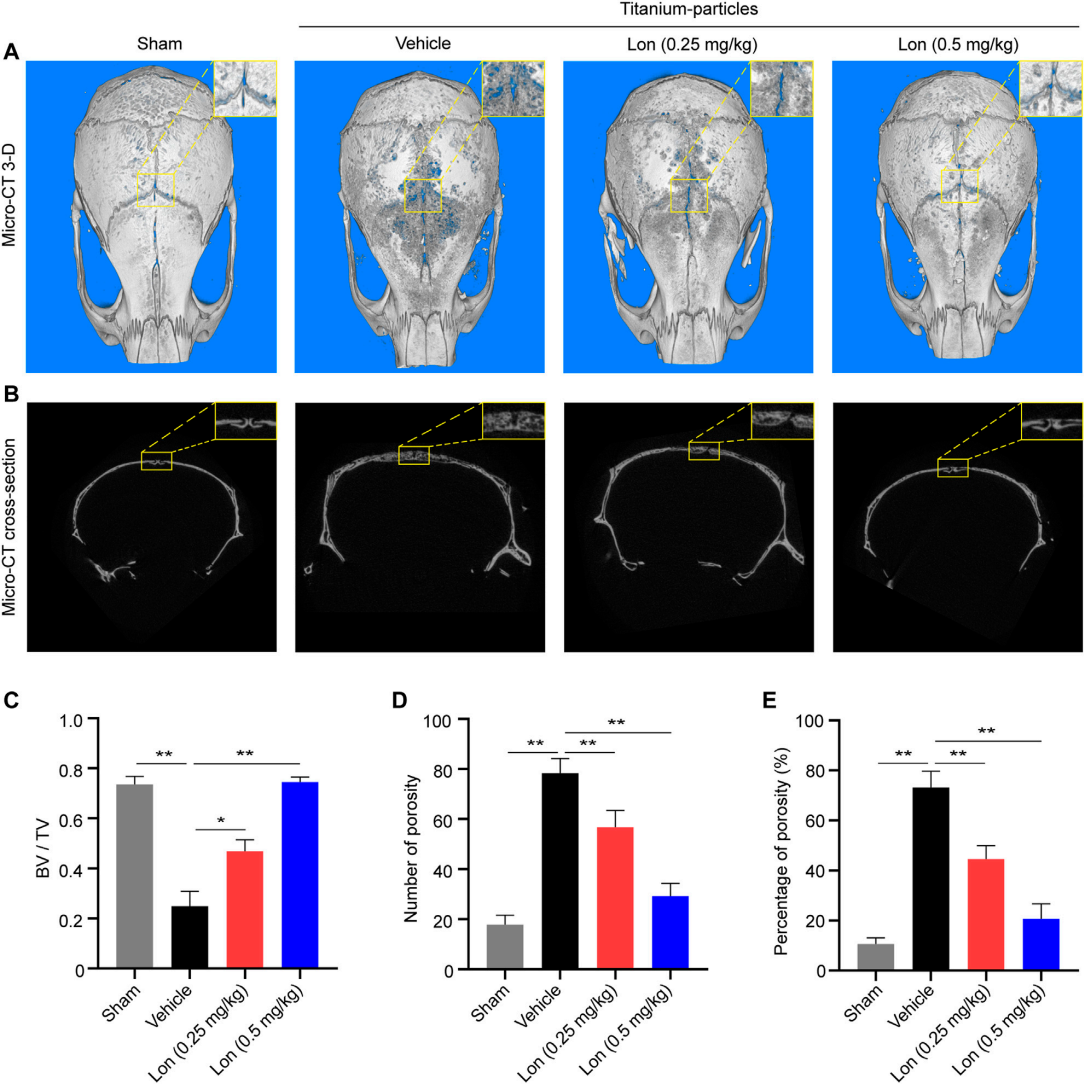
Lon prevents titanium particle-induced osteolysis and bone loss *in vivo*. **(A)** Representative Micro-CT 3-D constructional images showing that titanium particle-induced osteolysis was prevented by Lon administration. **(B)** Representative cross-section images showing that titanium particle-induced osteolysis was prevented by Lon administration. **(C)** BV/TV values **(D)** number of porosity and **(E)** percentage of porosity of ROI were quantified to assess bone microstructure. All of the above data are expressed as the mean ± SD, *n* = 5 per group. *p < 0.05, **p < 0.01 relative to Vehicle group. BV/TV, bone volume/tissue volume values; Lon, Lonafarnib; ROI, region of interest.

The given results indicated that Lon was a promising drug for inhibiting osteoclastic bone resorptive and osteoclastogenesis function *in vitro*, so we further evaluated its efficiency *in vivo*. A titanium particle-induced mouse calvarial osteolysis model was established, which is a common model to mimic periprosthetic osteolysis. 3-D constructional and cross-section images showed that titanium particles resulted in markedly lacunae in the Vehicle group as opposed to the Sham group, where Lon revised those erosions in a dosage-dependent way (Figures 3A,B). BV/TV of the ROI of calvaria was obviously enhanced by treatment with Lon (Figure 3C). 0.5 mg/kg dose Lon was more effective in terms of decreasing the number of porosity (Figure 3D) and percentage of porosity (Figure 3E). Lon was shown to be effective in preventing titanium particle-induced bone degradation.

### Lon Decreases Osteoclast Formation and Production of Pro-Inflammatory Cytokine *In Vivo*

**FIGURE 4:**
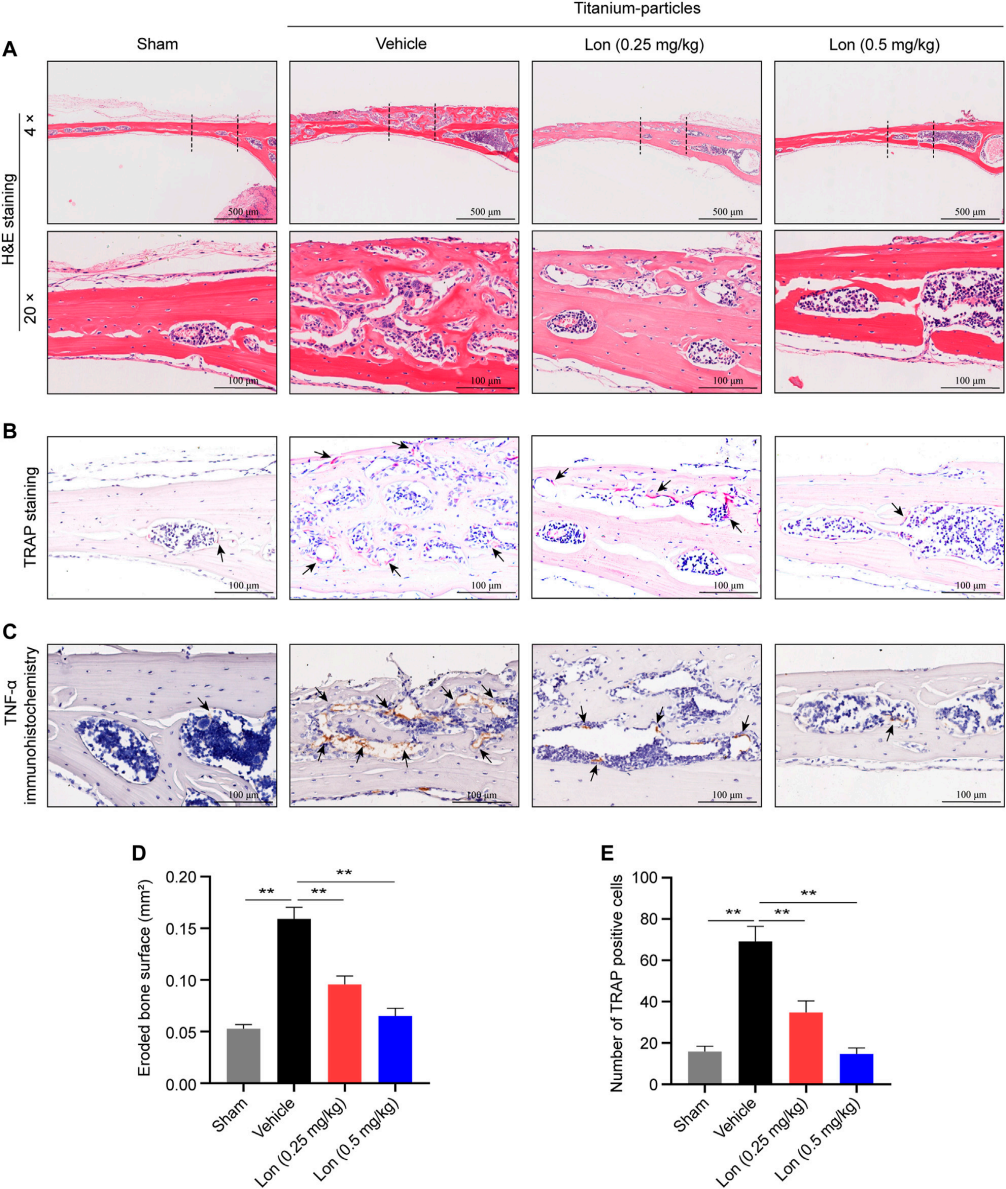
Lon decreases osteoclast formation and production of pro-inflammatory cytokine *in vivo.* **(A–C)** Representative images of histomorphometry of mouse calvaria staining with H&E, TRAP and TNF-α immunohistochemistry. **(D)** Quantitative analyses of eroded bone surface of ROI. **(E)** Quantitative analyses of numbers of TRAP-positive cells of ROI. All of the above data are expressed as the mean ± SD, *n* = 5 per group. *p < 0.05, **p < 0.01 relative to Vehicle group. H&E, hematoxylin and eosin; Lon, Lonafarnib; ROI, region of interest; TNF-α, tumor necrosis factor-alpha; TRAP, tartrate-resistant acid phosphatase.

Consistently, histological analysis demonstrated that Lon could reduce titanium particle-induced bone osteolysis (Figure 4A). H&E-stained section results further confirmed that the eroded bone surface was substantially elevated in the Vehicle group in comparison with the Sham group, and Lon had dose-dependently reduced the erosion (Figure 4D). TRAP staining examination illustrated that when in contrast with the Vehicle group, the proportion of TRAP-positive cells was significantly reduced when treatment with Lon, particularly in animals administered a 0.5 mg/kg dose (Figures 4B,E). As representative immunochemistry images shown in Figure 4C, tumor necrosis factor-alpha (TNF-α), a classical pro-inflammatory cytokine, appeared to be less staining after treatment with Lon compared with the Vehicle group. We also investigated interleukin-1 beta (IL-1β). The result showed that the Vehicle group presented more IL-1β-positive staining, while there was fewer staining after treating in Lon (Supplementary Figure S1). In conclusion, Lon inhibits osteoclast formation and production of pro-inflammatory cytokines *in vivo*.

### Lon Attenuates Relevant Osteoclastic Gene Expression

**FIGURE 5:**
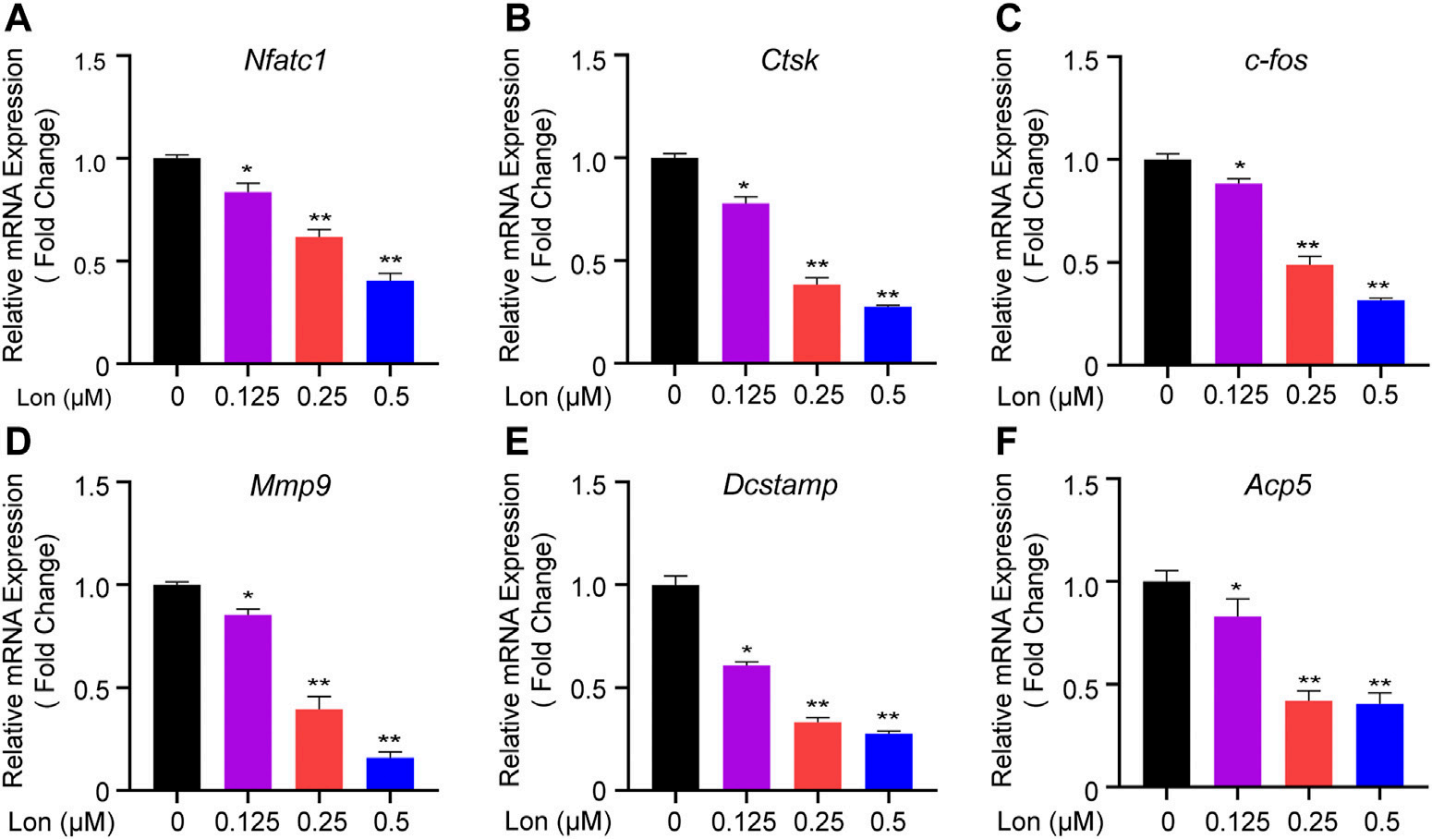
Lon attenuates relevant osteoclastic gene expression. **(A–F)** QT-PCR analysis of the expression levels of the relevant osteoclast genes *Nfatc1*, *Ctsk*, *c-fos*, *Mmp9*, *Dcstamp*, and *Acp5* relative to *β-actin* in BMMs stimulated with RANKL for 5 days in the presence of Lon. All of the above data are expressed as the mean ± SD, *n* = 3 per group. *p < 0.05, **p < 0.01 compared with control group (without Lon treatment). *Acp5*, acid phosphatase 5, tartrate resistant; *c-fos*, Proto-oncogene C-Fos; *Ctsk*, Cathepsin K; *Dcstamp*, dendritic cell-specific transmembrane protein; Lon, Lonafarnib; *Mmp9*, matrix metalloproteinase-9; *Nfatc1*, nuclear factor of activated T cells 1; RANKL, receptor activator of nuclear factor-κB ligand.

Relevant osteoclastic gene expressions were recorded during osteoclastogenesis. As shown in Figures 5A–F, several genes included *Nfatc1*, *Ctsk*, *c-fos*, *Mmp9*, *Dcstamp*, and *Acp5*, which specifically expressed in osteoclasts, were dose-dependently down-regulated by Lon treatment (0.125, 0.25, 0.5 μM). Those down-regulated genes indicated that Lon had a significant inhibitory effect during the process of osteoclastogenesis.

### Lon Suppresses NFATc1 Nuclear Translocation and Osteoclast-Relevant Protein Expression

**FIGURE 6:**
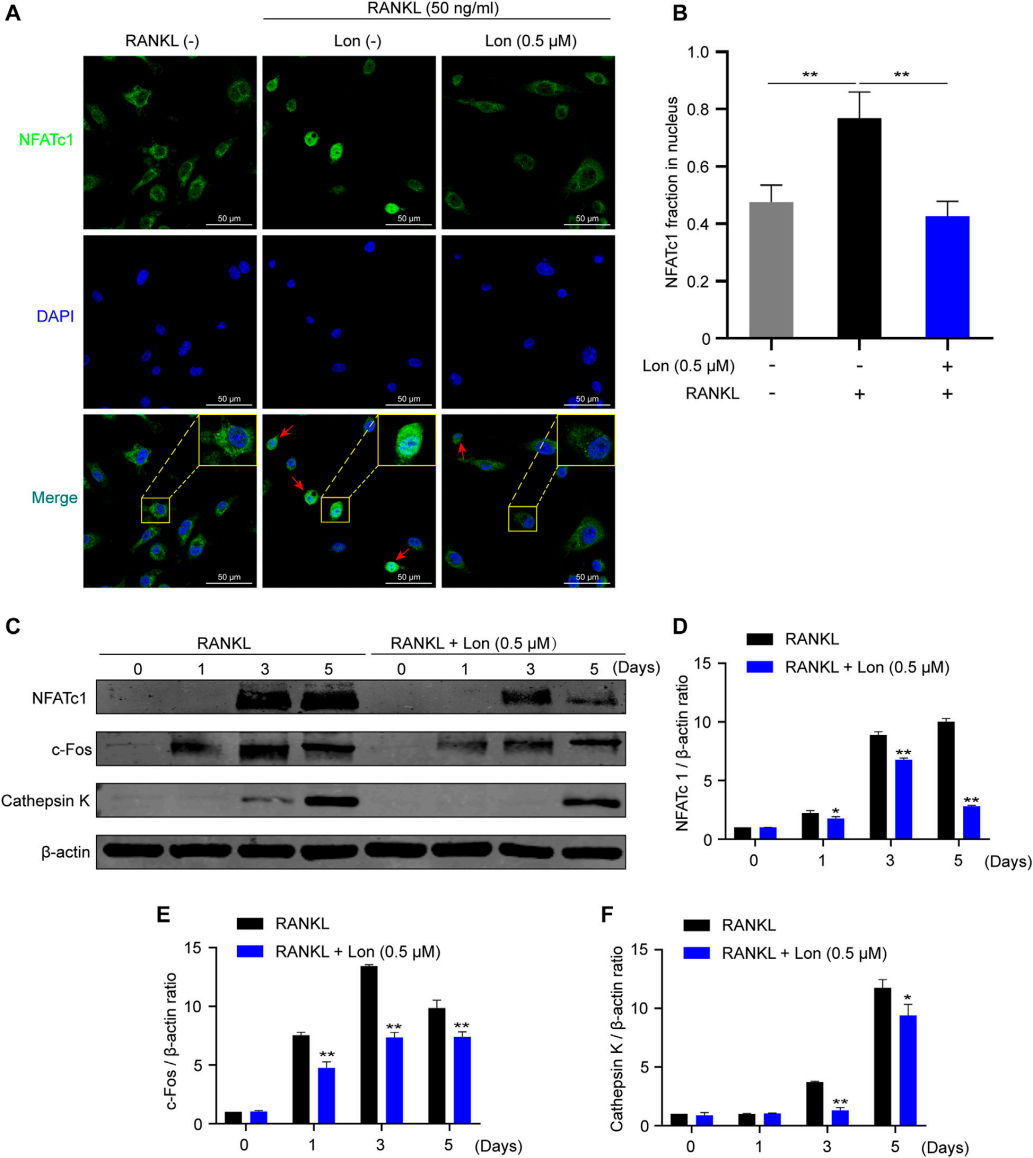
Lon suppresses NFATc1 nuclear translocation and osteoclast-relevant protein expression. **(A)** The suppressive effect of NFATc1 nuclear translocation treating with Lon was observed via NFATc1 immunofluorescence staining. **(B)** Relative NFATc1 fraction in nucleus was calculated through densitometric analyses (*n* = 4 per group). **(C)** Representative Western Blot images of the expression levels of NFATc1 and osteoclast-related proteins including c-Fos, and Cathepsin K during osteoclast formation. **(D–F)** Quantification of the ratios of band intensity of NFATc1, c-Fos, and Cathepsin K relative to *β-actin* (*n* = 3 per group). All of the above data are expressed as the mean ± SD. *p < 0.05, **p < 0.01 relative to RANKL-induced control group. BMMs, bone marrow macrophages; c-Fos, Proto-oncogene C-Fos; Lon, Lonafarnib; NFATc1, nuclear factor of activated T cells 1; RANKL, receptor activator of nuclear factor-κB ligand.

NFATc1 acts as a main transcriptional regulator during osteoclast differentiation and exhibits robust serine residues phosphorylation in the cytoplasm of the cells in a resting state (Park et al., 2018). NFATc1 is translocated into nuclei after dephosphorylation, and then modulates the expression of relevant osteoclast gene expression (Park et al., 2018). Hence, we investigated whether the restriction of NFATc1 nuclear translocation inhibited RANKL-induced NFATc1expression. As illustrated in Figure 6A, immunofluorescence images implied that the levels of NFATc1 in the nuclear fraction were elevated considerably following the activation of RANKL stimulation as opposed to the control, and Lon significantly reduced this elevation (Figure 6B). In summary, Lon could inhibit NFATc1 nuclear translocation.

Meanwhile, in line with the findings of the QT-PCR study, the results from western blot illustrated that the protein expressions of NFATc1, c-Fos, and Cathepsin K were dramatically attenuated following treatment with Lon (Figures 6C–F). Given that these downstream proteins are essential for osteoclast development and activity, Lon effectively provided the anti-osteoclast effect.

### Lon Dampens FTase and Inhibition of FTase Reduces Osteoclast Activation

**FIGURE 7:**
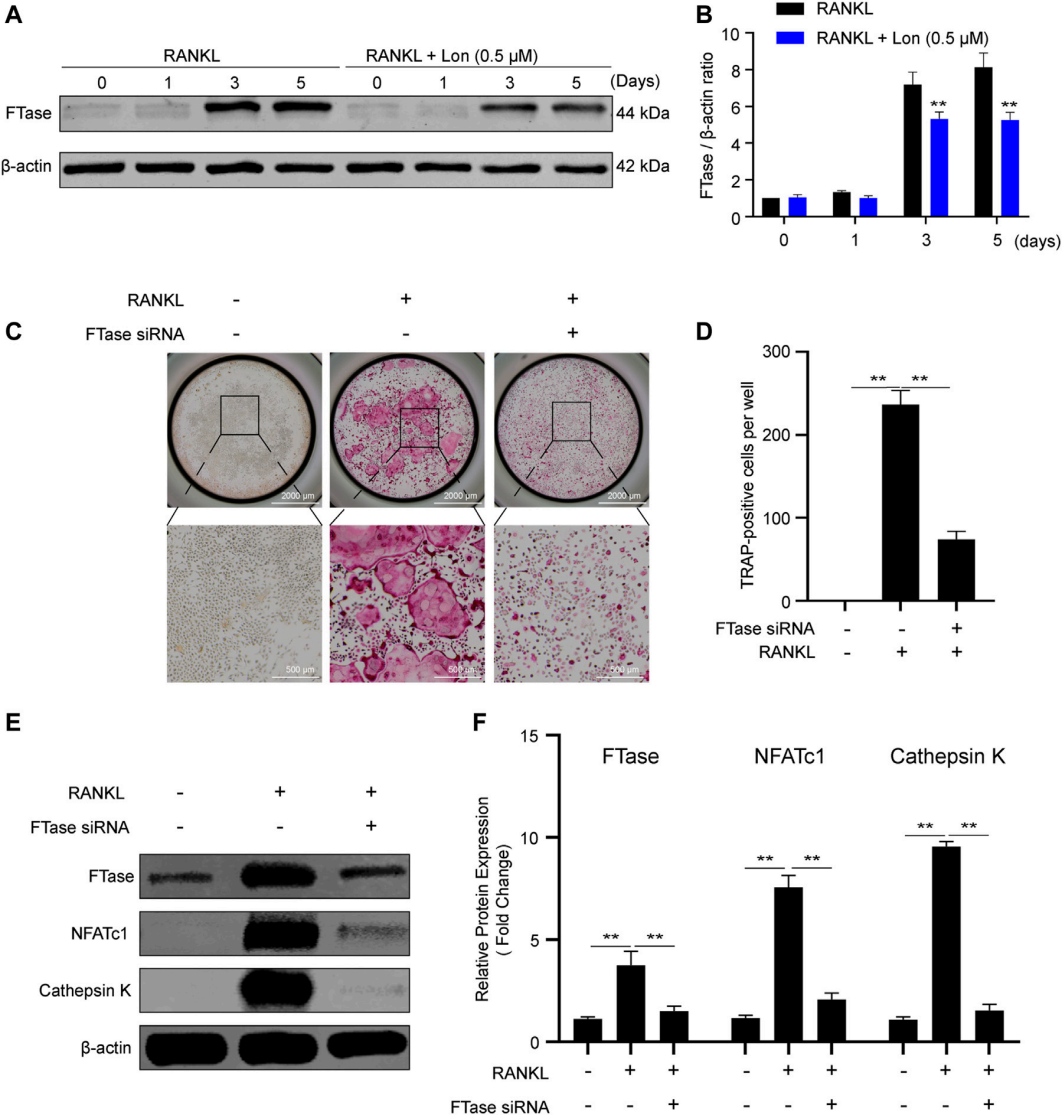
Lon dampens FTase and inhibition of FTase reduces osteoclast activation. **(A)** FTase expression and the effect of Lon on suppressing FTase was assessed via Western blotting at the indicated time points during osteoclastogenesis. **(B)** Quantification analysis of the ratio of FTase to β-actin. **(C)** Representative images of TRAP staining showing that the effects of RANKL-induced osteoclastogenesis via silence the FTase gene. **(D)** Quantification analysis of TRAP-positive cells. **(E)** Representative Western Blot images of the expression levels of FTase and osteoclast-related proteins including NFATc1 and Cathepsin K through silence the FTase gene. **(F)** Quantification of the ratios of band intensity of FTase, NFATc1, and Cathepsin K relative to β-actin (*n* = 3 per group). All of the above data are expressed as the mean ± SD. *p < 0.05, **p < 0.01 relative to RANKL-induced control group. FTase, farnesyltransferase; Lon, Lonafarnib; NFATc1, nuclear factor of activated T cells 1; RANKL, receptor activator of nuclear factor-κB ligand.

To find out the role of FTase in osteoclasts, we investigated FTase expression during osteoclastogenesis. As shown in Figures 7A,B, FTase expression rose with osteoclast activation, and Lon significantly inhibited those elevations by suppressing osteoclast differentiation. The results indicated that FTase might perform an instrumental function during this process. We employed FTase siRNA to knock down the endogenous FTase α-subunit expression and evaluated its impacts on RANKL-stimulated osteoclastogenesis to thoroughly demonstrate the function performed by FTase-dependent in the growth of osteoclasts. 80–90% of BMMs were transfected. In contrast with NC siRNA, FTase siRNA dramatically lowered the number of TRAP-positive cells (Figures 7C,D). Additionally, FTase siRNA reduced endogenous FTase expression (Figures 7E,F). Meanwhile, osteoclast-relevant proteins, including NFATc1 and Cathepsin K were significantly decreased by FTase siRNA (Figures 7E,F). In summary, the results demonstrated that Lon inhibits FTase to suppress RANKL-induced osteoclast activation.

### Lon Inhibits the ERK Pathway Activation in the Process of RANKL-Induced Osteoclastogenesis

**FIGURE 8:**
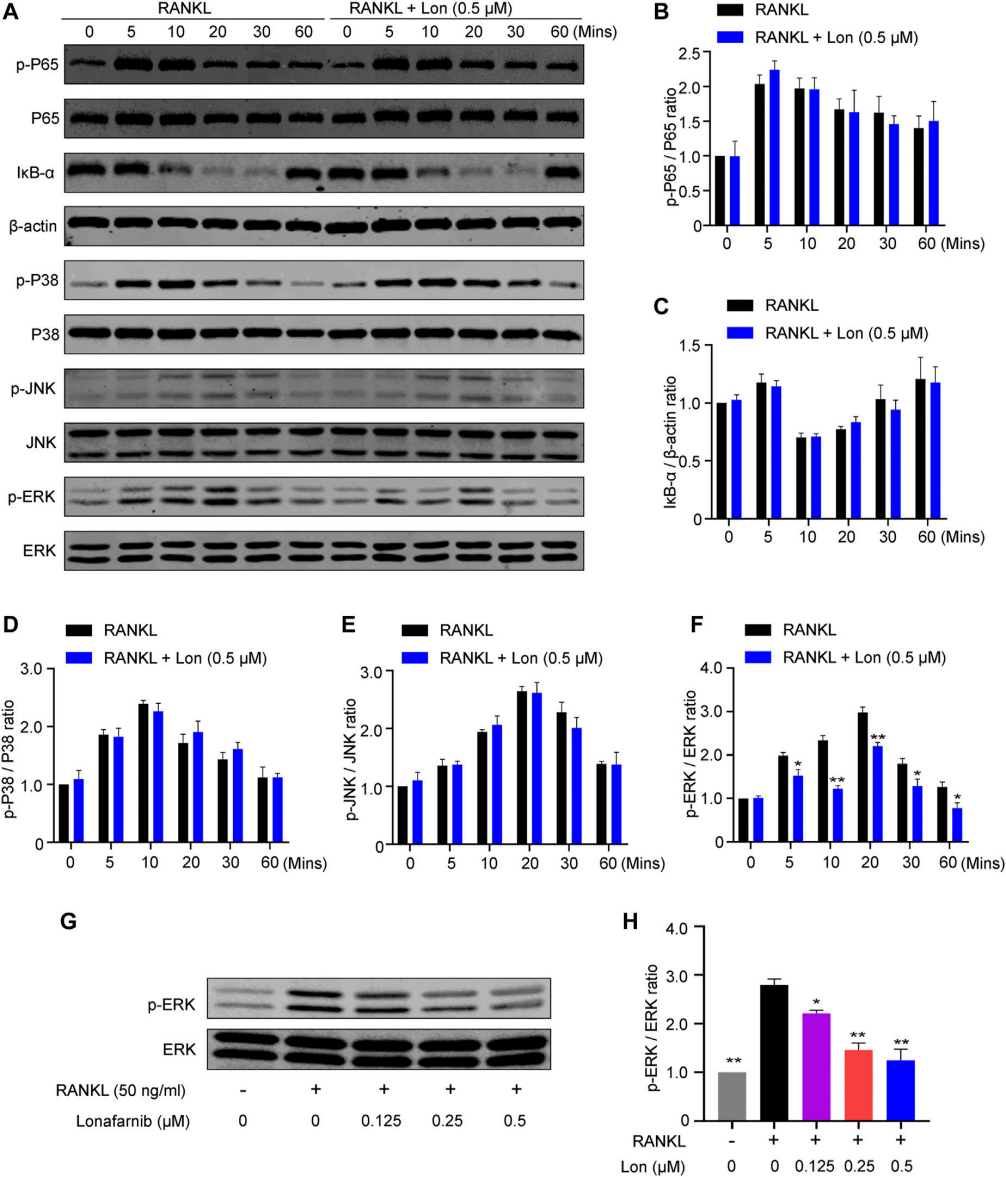
Lon inhibits the activation of ERK pathway during RANKL-induced osteoclastogenesis. **(A)** Representative Western blot images of the effect of Lon on RANKL-induced P65 protein phosphorylation, IκB-α degradation, P38 protein phosphorylation, JNK protein phosphorylation, and ERK protein phosphorylation. **(B–F)** Quantification analysis of the ratio of p-P65 to P65, IκB-α to β-actin, p-P38 to P38, p-JNK to JNK, and p-ERK to ERK (*n* = 3 per group). **(G)** Representative Western blot images of the effect of different concentration of Lon on RANKL-induced ERK protein phosphorylation. **(H)** Quantification of the ratio of p-ERK to ERK treated with different concentration of Lon. All of the above data are expressed as the mean ± SD, *n* = 3 per group. *p < 0.05, **p < 0.01 relative to RANKL-induced control group. BMMs, bone marrow macrophages; Lon, Lonafarnib; RANKL, receptor activator of nuclear factor-κB ligand.

To delve into the mechanism by which Lon inhibited osteoclast development and activity, a western blot analysis was performed to discover whichever pathway was blocked by Lon. BMMs were cultured in a medium that contained 50 ng/ml RANKL for the stated time (0, 5, 10, 20, 30, and 60 min) after treatment with Lon for 2 h. The findings illustrated that the activation of the NF-κB signaling pathway was not affected by Lon (Figures 8A–C). Moreover, augmented phosphorylation of P38 and JNK protein wasn’t significantly different in the presence or absence of Lon (Figures 8A,D,E). However, Lon has significantly inhibited the phosphorylation of ERK protein, especially at 10 min (Figures 8A,F). To further explore the effect of Lon on the phosphorylation of ERK protein, we stimulated BMMs with various concentrations of Lon for 2 h. Subsequently, we treated them for 10 min using RANKL. The findings revealed that Lon inhibited the ERK protein phosphorylation in a dosage-dependent manner (Figures 8G,H).

## Discussion

For patients with end-stage joint disease, TJA is the procedure that has had the most success rate in recent years. However, AL accompanied by PPOL is one of the main factors that resulted in TJA failure (Abu-Amer et al., 2007). The mechanism of wear particle-induced periprosthetic osteolysis has not been fully understood to date, osteoclast-mediated bone resorption has been identified as the primary driver for this process (Masui et al., 2005; Goodman and Gallo, 2019; Zhang et al., 2020). Suppression of osteoclastic activation is a key to preventing wear-particles induced PPOL (Goodman and Gallo, 2019; Zhang et al., 2020). At present, there are no perfect non-operative interventions for PPOL. We hypothesized that bisphosphonates have some benefit in preventing unfavorable mechanically-based bone remodeling related to the placement of a prosthesis; nevertheless, the application of bisphosphonates in treating particle-associated PPOL has not gained wide attention (Wilkinson and Little, 2011). Inhibition of osteoclast activity and function has been shown with denosumab; nonetheless, there is little evidence of its effectiveness in slowing the growth of osteolysis and aseptic loosening in TJA (Skoldenberg et al., 2016). Additionally, numerous adverse reactions are correlated with these drugs, such as gastrointestinal reactions, allergies, headaches, and nausea. We are in actual need of a drug that is efficacious in treating PPOL while not causing major side effects. In this study, we were able to confirm that Lon has the ability to prevent PPOL.

Osteoclasts have been demonstrated to be the only kind of cell with bone-resorption function (Uehara et al., 2018). The quantity, function, and survival of osteoclasts at a specific location inside a bone multicellular unit determines the degree of bone resorption (Goodman and Gallo, 2019). It’s essential to suppress the activation of osteoclast after TJA. In the present research, we discovered that Lon inhibited the formation of osteoclasts in a dosage-dependent way under 1 μM without cytotoxicity. Excessive bone degradation occurs as a consequence of increased bone resorption; consequently, limiting osteoclast bone resorption activity is essential to the creation of antiresorptive drugs in the future (Zheng et al., 2019). Several studies concluded that inhibition of osteoclastic acidification leads to inhibition of bone resorption (Karsdal et al., 2005; Qin et al., 2011). We investigated the osteoclastic characteristics of Lon and discovered that it strongly suppressed acidification of the resorption lacunae, culminating in a very powerful suppression of bone resorption. The promising findings showed that Lon might serve as a possibly innovative treatment for osteoclast-related PPOL in the future.

After that, a PPOL model of titanium particle-induced mouse calvarial osteolysis was introduced to evaluate the therapeutic impacts of Lon *in vivo*. Titanium particles, which are one type of wear particles and located between the implant and the bone, could augment the aggregation of BMMs (Yuan et al., 2017), promote the BMMs differentiation into osteoclasts, and the release of vast pro-inflammatory components such as IL-1β and TNF-α. Thus, titanium particles are widely used in a calvarial osteolytic model (Shi et al., 2020; Li et al., 2021). Our micro-CT scanning and histological analysis indicated that titanium particles resulted in calvarial surface erosion and Lon significantly ameliorated the erosion. In this research, we discovered that Lon therapy reduced the production of osteoclasts near the interface of the eroded region in the presence of calvarial osteolysis using TRAP staining *in vivo*. Moreover, Lon had a substantial inhibition activity on the release of pro-inflammatory factors. In general, Lon protects osteolysis *in vivo*.

As a transcription factor, NFATc1 attracts extensive attention for its role in osteoclast differentiation and development. It is reported to trigger a number of signaling pathways that are implicated in osteoclastogenesis (Aliprantis et al., 2008). The capacity to stimulate transcription is lost once NFATc1 is evenly distributed throughout the cytoplasm. Translocation of NFATc1 into the nucleus, on the other hand, results in the possibility for it to trigger transcription (Masuda et al., 1998). While the activated NFATc1 induces the expression of multiple osteoclast-specific genes, such as *Acp5*, *Mmp9*, *Dcstamp,* and *Ctsk*, NFATc1 also acts on itself to auto-amplify the NFATc1-mediated transcriptional program (Takayanagi et al., 2002). In the present study, we found that Lon not only inhibited RANKL-induced NFATc1 nuclear translocation but also reduced the expression at the protein and the mRNA levels. However, the relevant mechanism of the suppression of NFATc1 still remains unknown. It would be triggered by direct interference of NFATc1 activation or the indirect inhibition of upstream ERK signaling. C-Fos, which is another master transcription factor involving osteoclast differentiation and coordinates with NFATc1 in the case of activator protein 1 (AP-1) (Takayanagi et al., 2002), was significantly inhibited by the treatment of Lon.

M-CSF stimulates the development as well as the survival of cells inside the monocyte/macrophage lineage, particularly osteoclasts, which are multinucleated cells that are formed to achieve bone resorption (Boyle et al., 2003). The musculoskeletal system is frequently affected by the M-CSF/Ras/MAPK or M-CSF/Ras/PI3K pathway, implying that the M-CSF/Ras pathway activation influences the cell that modulates bone formation and homeostasis (Bradley et al., 2008; Stevenson et al., 2011). Tsubaki et al. reported (Tsubaki et al., 2012) that blocking the Ras/ERK pathway enhanced osteoprotegerin expression and inhibited the RANKL, M-CSF, and CD9 expression in the bone marrow-derived stromal cell line ST2. FTase performs a function in the targeting of Ras. FTase promotes the stimulation of Ras families in order to facilitate the processing of Ras proteins post-translation (Delarue et al., 2007). Cancer markers such as tumor progression, recruitment of tumor regression, stoppage of mitosis at prometaphase, induction of apoptosis, angiogenesis, and suppression of anchorage-dependent and -independent growth have all been demonstrated to be attenuated by inhibiting FTase (Klochkov et al., 2019). In the present research, we discovered that FTase was upmodulated after RANKL stimulation, suggesting it may perform an instrumental function during RANKL-induced osteoclastogenesis. Hence, we treated osteoclasts with FTase inhibitor Lon and FTase siRNA. The results indicated that inhibition of FTase significantly decreases the relevant protein expressions and the activation of osteoclasts, thereby suppressing osteoclast bone resorption function.

Finally, we explored the underlying mechanisms of Lon in inhibiting osteoclast formation and function. RANKL signal transduction is closely related to osteoclast formation. RANKL through binding cellular receptor RANK activates downstream signaling pathways that control osteoclastic differentiation, resorption triggered by mature osteoclasts, as well as their survival and functions in additional rounds of bone deterioration at nearby locations (Boyle et al., 2003). MAPK proteins include three major MAPK family members (P38, JNK, and ERK). MAPK kinases are responsible for the activation of MAPKs via phosphorylation of threonine and tyrosine while MAPK phosphatases (MKPs) inactivate the MAPK via dephosphorylation (Koga et al., 2019). Normal osteoclast differentiation and activation stimulated by RANKL depends on the signal activity of MAPK pathway (Boyle et al., 2003). ERK is essential for the survival of osteoclasts (Lee et al., 2001; Ouyang et al., 2014). ERK pathway participates in the negative modulation of osteoclastogenesis (Boyle et al., 2003). Engsig et al. (2000) reported that ERK activation can enhance the expression of Matrix metalloproteinase 9 (MMP-9), which may stimulate the migration of osteoclasts and the resorption of bone. In general, our data demonstrated that Lon was able to attenuate RANKL-induced MAPK signaling by inhibiting ERK activation, although it had no effect on the NF-κB pathway signaling.

In recent years, more studies focused on the role of bone formation in wear debris-induced osteolysis. A reduction of bone forming ability may aggravated osteolysis (O'Neill et al., 2013). Impairment of osteoprogenitors may further affect bone formation during osteolysis (Goodman et al., 2006). Pro-inflammatory cytokine TNF-α independently suppressed procollagen α1 mRNA expression and subsequent osteoblast collagen synthesis (Lee et al., 2012; Wang and He, 2020). The down regulation of WNT and BMP signaling pathways suppressed osteogenic activity during titanium particle-induced osteolysis (Nam et al., 2017). Alleviated PPOL may be benefited from the promotion of bone formation. Future research regarding the effects of Lon on bone formation is thus warranted.

**FIGURE 9:**
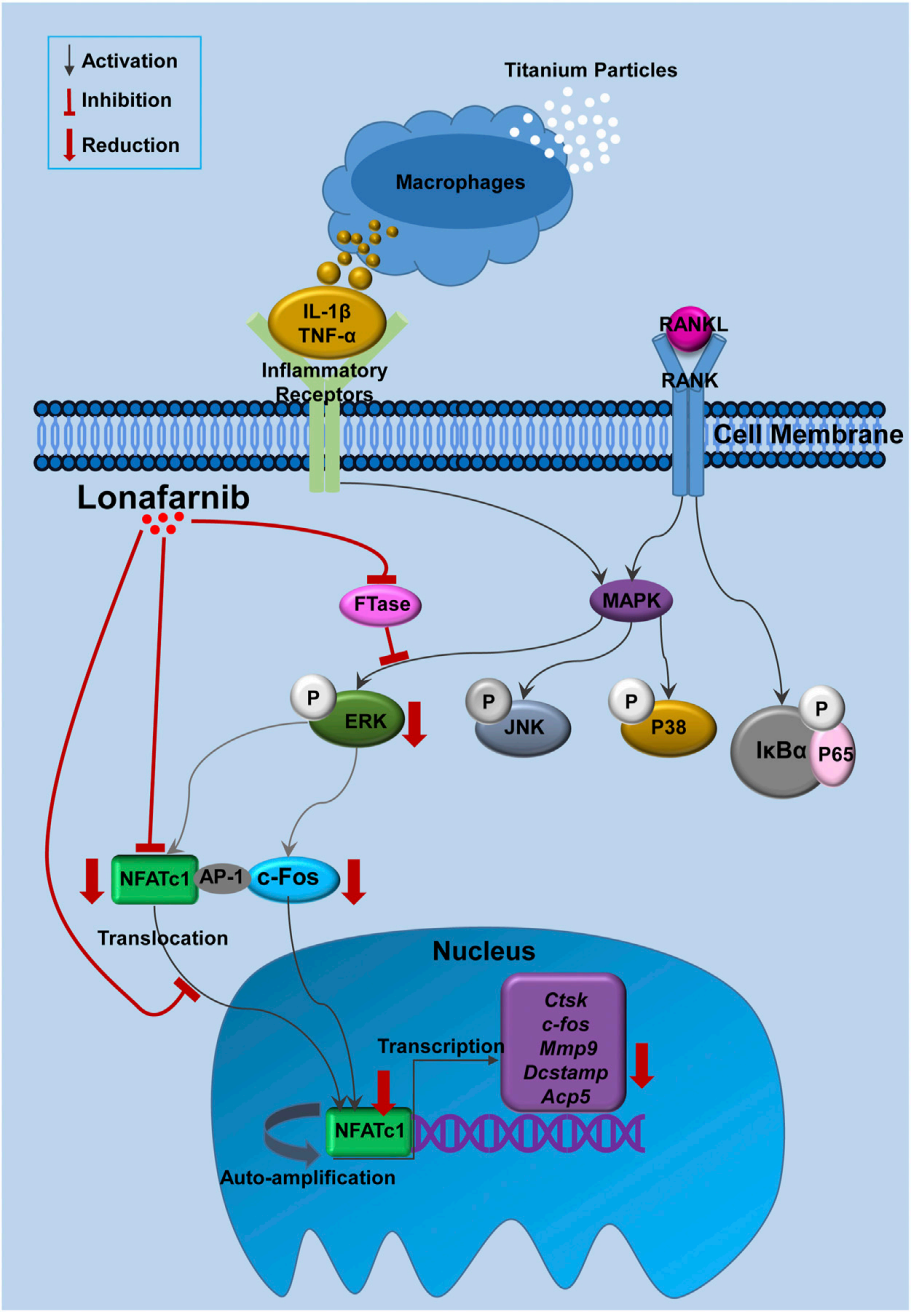
Proposed scheme for Lon suppression of osteoclast production. Titanium particles stimulate macrophages and other cells to release pro-inflammatory substances (such as IL-1β and TNF-α) which bind to the inflammatory receptors or increase osteoclast-related cytokines, such as RANKL. When RANKL binds to RANK, NF-κB and MAPKs (including P38, JNK and ERK) pathways are activated, following the nuclear translocation and auto-amplification of NFATc1. As a result, the osteoclast-relevant genes such as *Ctsk*, *c-fos*, *Mmp9*, *Dcstamp*, and *Acp5* are upregulated. Our results demonstrate for the first time that Lon inhibited FTase to attenuate the activation of ERK pathways, subsequently resulted in decreasing of NFATc1 as well as its downstream gens. *Acp5*, acid phosphatase 5, tartrate resistant; AP-1, activator protein 1; *c-fos*, Proto-oncogene C-Fos; *Ctsk*, Cathepsin K; *Dcstamp*, dendritic cell-specific transmembrane protein; FTase, farnesyltransferase; IL-1β, interleukin-1 beta; Lon, Lonafarnib; *Mmp9*, matrix metalloproteinase-9; NFATc1, nuclear factor of activated T cells 1; RANKL, receptor activator of nuclear factor-κB ligand; TNF-α, tumor necrosis factor-alpha.

In conclusion, this study has verified for the first time that Lon inhibited FTase to attenuate the activation of ERK pathways, subsequently resulting in the decrease of NFATc1 and its downstream genes (Figure 9). When these signaling events occur *in vitro*, they lead to the reduction of osteoclast production and bone resorption function. In addition, Lon was demonstrated to inhibit titanium particles-induced osteolysis *in vivo* via decreasing pro-inflammatory cytokine production. Therefore, Lon may serve as a viable alternative candidate in PPOL or other illnesses correlated with inflammation and overactive osteoclasts.

## Data Availability

The original contributions presented in the study are included in the article/Supplementary Material, further inquiries can be directed to the corresponding authors.
