# Supplementary material for: Lonafarnib Inhibits Farnesyltransferase via Suppressing ERK Signaling Pathway to Prevent Osteoclastogenesis in Titanium Particle-Induced Osteolysis
Source: Front Pharmacol. 2022 Mar 1;13:848152. doi: 10.3389/fphar.2022.848152 (PMC8921770; doi:10.3389/fphar.2022.848152)
Supplement: Supplementary file 1 [file DataSheet1.PDF]

Supplementary Material

Supplementary Figures and Tables

Supplementary Figures

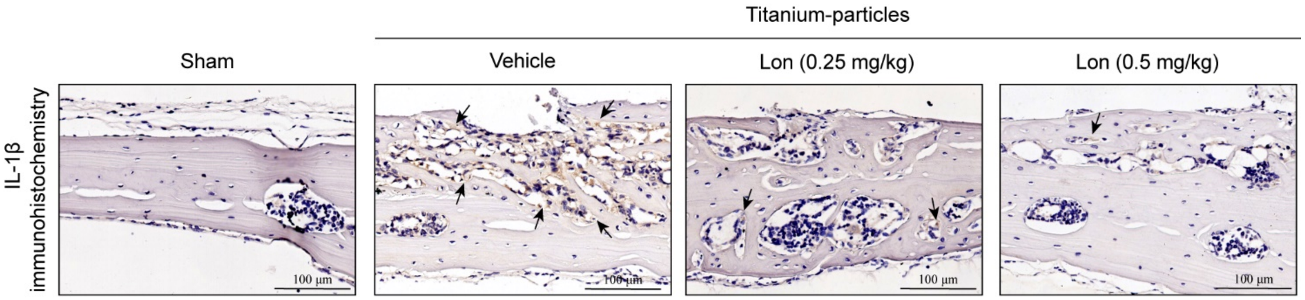

Supplementary Figure S1. Lon decreases the production of IL-1β i*n vivo.* Representative images of histomorphometry of mouse calvaria staining with IL-1β immunohistochemistry. IL-1β, interleukin-1 beta; Lon, Lonafarnib.
